# Continuous nicotine exposure does not affect resurgence of alcohol seeking in rats

**DOI:** 10.1371/journal.pone.0202230

**Published:** 2018-08-15

**Authors:** Charles C. J. Frye, Jillian M. Rung, Rusty W. Nall, Ann Galizio, Jeremy M. Haynes, Amy L. Odum

**Affiliations:** Department of Psychology, Utah State University, Logan, Utah, United States of America; Radboud University Medical Centre, NETHERLANDS

## Abstract

Alcohol is the most commonly used drug in the United States and alcohol abuse can lead to alcohol use disorder. Alcohol use disorder is a persistent condition and relapse rates following successful remission are high. Many factors have been associated with relapse for alcohol use disorder, but identification of these factors has not been well translated into preventative utility. One potentially important factor, concurrent nicotine use, has not been well investigated as a causal factor in relapse for alcohol use disorder. Nicotine increases the value of other stimuli in the environment and may increase the value of alcohol. If nicotine increases the value of alcohol, then nicotine use during and after treatment may make relapse more probable. In the current study, we investigated the effect of continuous nicotine exposure (using osmotic minipumps to deliver nicotine or saline, depending on group, at a constant rate for 28 days) on resurgence of alcohol seeking in rats. Resurgence is a type of relapse preparation that consists of three phases: Baseline, Alternative Reinforcement, and Resurgence Testing. During Baseline, target responses produced a dipper of alcohol. During Alternative Reinforcement, target responses were extinguished and responses on a chain produced a chocolate pellet. During Resurgence Testing, responses on the chain were also extinguished and a return to responding on the target lever was indicative of resurgence. Multilevel modeling was used to analyze the effect of nicotine on resurgence. Both the nicotine and saline group showed resurgence of alcohol seeking, but there was no difference in the degree of resurgence across groups. Future directions could involve testing alternative drug delivery techniques.

## Introduction

Alcohol is the most commonly used drug in the United States [1]. Alcohol is a depressant and is rewarding due to its disinhibiting and euphoria-producing effects. Due to the high reinforcing efficacy of alcohol, some people are unable to moderate the frequency and/or intensity of their drinking and develop alcohol use disorder (AUD). AUD has a negative effect on a person’s ability to thrive in society and is associated with long-term health detriments. Alcohol is responsible for an average of 88,000 deaths per year and the effects of alcohol use cost the United States $220 billion each year [2]. A recent study, using a large representative sample, found that 13.9% of individuals met the criteria for AUD in the last year and 29.1% of individuals met the criteria for AUD at some point in their life [3]; these numbers indicate a significant increase over the past decade [4].

The rate of relapse for AUD following remission is high [5], but the reasons for high rates of relapse are not well-understood. Relapse for AUD, following successful treatment, has been linked to a variety of social and biological markers, but these findings have not been well translated into preventative utility. Indeed, most people relapse at least once before successfully overcoming the disorder [6]. One under-investigated factor for the high rates of relapse in people who are in remission for AUD is concurrent nicotine use.

Tobacco cigarette consumption is still a leading cause of preventable death in the United States [7] and electronic cigarette use is on the rise. Nicotine is the constituent in tobacco cigarettes that is believed to be responsible for the high rates of addiction. Dependence is more common with nicotine than with any other substance [8]. Although tobacco cigarette consumption has declined in recent years, alternative forms of nicotine delivery have increased [1,9]. The majority of past research on nicotine use has focused on the deleterious health effects of tobacco cigarette consumption. Whereas much is known about the effects of tobacco cigarettes on health, relatively little is known about the behavioral effects of nicotine consumption alone.

Nicotine is a complex drug of abuse. According to the dual reinforcement model of nicotine action [10], nicotine consumption has both primary reinforcing effects and reward-enhancing effects. As a primary reinforcer nicotine is relatively weak [11], but is a much stronger reinforcer if it is accompanied by other stimuli. Nicotine increases the value of these stimuli through its reward-enhancing properties and increases the behavior that produces them. Furthermore, nicotine ingestion has been shown to increase the value of other stimuli in the environment that are unrelated to nicotine delivery. For example, nicotine increases the value of food [12], contingent light presentations [13], sucrose [14], attractiveness to facial cues [14], reported happiness while watching films categorized as “happy films” [15], and sensory rewards such as music [16]. It has been argued that the reward-enhancing properties of nicotine are, at least partially, responsible for the prevalence of its use [8,14].

Nicotine abuse is often comorbid with alcohol abuse and may facilitate relapse for AUD symptoms. Approximately 80–95% of people with alcoholism smoke tobacco cigarettes [17]. Selective breeding for high alcohol preference in mice simultaneously increases sensitivity to nicotine’s reinforcing effects [18]. In rats, exposure to nicotine increases alcohol consumption [19]. The increase in alcohol consumption under the influence of nicotine could be the result of nicotine increasing the value of alcohol and its corresponding effects. The increase in the value of alcohol–through nicotine’s reward-enhancing properties–may lead to higher rates of relapse for those undergoing treatment for alcoholism if they continue to use nicotine during and after treatment. Human clinical observations support this assertion. Female smokers who undergo treatment for alcoholism have higher cravings for alcohol than their non-smoking counterparts [20]. Daily smoking abstinence is associated with lower alcohol consumption, lower urges to drink, greater alcohol abstinence self-efficacy, and perceived self-control demands [21]. Furthermore, smoking during abstinence for alcohol, when people are in treatment for AUD, is associated with an increase in the frequency of urges to drink [22,23]. In physiological studies, nicotine has been found to increase salivary cortisol levels, which are associated with relapse [24] and promote sustained GABA_A_ receptor levels, which are associated with craving for alcohol [25]. The reward-enhancing properties of nicotine could be (at least partially) responsible for the high rates of relapse seen in those with AUD, due to the high rate of concurrent nicotine use in this population. The causal relation of nicotine exposure to relapse for alcohol seeking is difficult to study, however, in human populations.

Animal models of relapse provide a methodology for assessing the effect of nicotine on relapse for alcohol seeking. There are several ways to model relapse in the laboratory (e.g., spontaneous recovery, reinstatement, renewal, resurgence, etc.) [26]. Each of these methodologies share the same overarching research strategy. For example, each relapse preparation consists of Phase 1: acquisition of target responding (e.g., responding on a lever to earn a drug), Phase 2: the cessation/reduction of target responding (e.g., no longer responding on the lever that is associated with drug), and Phase 3: a relapse test (e.g., some manipulation occurs to assess whether target responding recurs). However, the strategies employed during Phase 2 and Phase 3 set the relapse methodologies apart. The key features of resurgence, one type of relapse methodology, offer promise as a human analogue of relapse [27].

The resurgence paradigm models acquisition (e.g., of drug use or another problem behavior), cessation (through alternative reinforcement that is incompatible with the problem behavior), and relapse (through removal of alternative reinforcement) of problem behavior [28]. In animal models, these processes are modeled by making a reward (e.g., a drug) available for responding on a target manipulandum (e.g., lever) during a baseline phase. Once responding is established and the subject reliably earns rewards, target responses are placed on extinction and responses to an alternative manipulandum (e.g., a chain) produce an alternative reward. Finally, once responding on the target manipulandum has stabilized in the presence of the alternative manipulandum and its associated reward, responses on the alternative manipulandum are also placed on extinction and a return to the target manipulandum is indicative of relapse (in this case, resurgence).

Resurgence is an especially attractive model of relapse because it adequately captures the process of problem behavior acquisition, treatment, and potentially relapse (upon treatment termination) in the real world [28]. For example, a person acquires drug-taking when they encounter the reinforcing effects of the drug and begin using the drug regularly. In severe cases, the person cannot moderate use of the drug and must receive help from a treatment facility. Inside the treatment facility, drugs are no longer available and we can bring them in contact with alternative sources of reinforcement (e.g., social reinforcement, hobbies, etc.). Finally, when they check out of the treatment facility, those alternative sources of reinforcement are no longer available, and they may return to using drugs (i.e., they may experience resurgence of drug taking). Thus, this methodology captures the key features of acquisition, treatment, and relapse for severe problem behavior [27]. Despite the attractive features of resurgence as an analogue to severe human problem behavior, it is not as widely used as other relapse techniques (e.g., reinstatement).

To assess the role of nicotine in relapse for AUD symptomology, we conducted an experiment assessing the effect of continuous nicotine exposure on resurgence for alcohol seeking in rats. First, rats acquired alcohol consumption in their home cage. Next, the rats responded on levers to earn alcohol rewards in an operant chamber. Then, we conducted surgery on each subject to implant an osmotic minipump that delivered saline or nicotine (depending on the group) at a constant rate for 28 days. Osmotic minipumps were chosen over pre-session drug injections because injections can cause stress [29], which itself can induce relapse in rats [30]. Finally, all subjects experienced a typical resurgence task to model what humans experience in the clinic: a drug-taking phase (Baseline), a treatment phase (Alternative Reinforcement), and a relapse phase (Resurgence Testing). We hypothesized that both groups would show relapse (increased responses on the lever that was associated with alcohol rewards, the target lever) during resurgence testing, but the nicotine group would relapse to a greater extent.

## Materials and methods

### Subjects

Twelve experimentally naïve male Long Evans rats, aged 71–90 days, were obtained from Charles River Laboratories. Following Pretraining (see below), rats were assigned to one of two groups: Sal (saline; *n* = 6) or Nic (nicotine; *n* = 6), such that groups were matched based on dose of self-administered alcohol during pretraining. Rats were maintained at 100% of their free-feeding body weight through post-session supplemental feeding throughout the experiment. Rats were fed LabDiet^®^ rat chow and had continuous access to water in their home cages. Rats were individually housed in a temperature-controlled colony room with a 12:12 hour light/dark cycle. Sessions were conducted every day at approximately the same time each day during the light cycle. The current study was approved by the Utah State University Institutional Animal Care and Use Committee.

### Materials

#### Apparatus

Four standard Coulbourn operant chambers (Coulbourn Instruments) enclosed in light- and sound-attenuating cubicles were used for this experiment. Each operant chamber was equipped with two fixed levers on the front panel. The lever designated as the target response (i.e., left or right) was counterbalanced across rats. Each lever had a green, red, and yellow LED light above the lever. A receptacle was located in the middle of the front panel and equipped with a light. A pellet dispenser above the receptacle delivered 45-mg dustless precision chocolate pellets into the receptacle. Chocolate pellets were obtained from Bio-Serv^®^. There was also a dipper located under the receptacle that could be raised to provide 0.1 mL of liquid solution. Each chamber had a houselight located on the ceiling of the front panel to provide general illumination.

#### Drugs

Distilled water and 95% ethanol were used to make a 20% ethanol solution that was self-administered orally in the home cage during the two-bottle choice procedure and in the operant chamber. During surgery, osmotic mini-pumps were filled with 2 mL of drug solution. The drug solution used for the Sal group was 0.9% sterile saline solution. The drug solution for the Nic group was made using nicotine hydrogen tartrate salt (MP Biomedicals, LLC.) dissolved in 0.9% sterile saline solution. Nicotine was delivered at approximately 3 mg/kg/day [12].

### Procedure

#### Two-bottle choice

An intermittent-access two-bottle choice procedure was used to establish ethanol consumption [31]. Rats were given access to a bottle containing 20% ethanol solution 3 days per week (i.e., Monday, Wednesday, Friday, or Tuesday, Thursday, Saturday) in their home cages. Water was freely available in another bottle during ethanol sessions and ethanol-free sessions. This phase lasted for 8 weeks (24 sessions) and all subjects consumed a dose of alcohol that was above the criterion dose (> 0.3 g/kg) [31] by the end of this phase.

#### Pretraining

Following the two-bottle choice procedure, rats began daily sessions in the operant chambers. Session initiation consisted of illumination of the houselight and the target lever stimulus light. Rats initially responded on the target lever according to a fixed ratio (FR) schedule of reinforcement. Reinforcer deliveries consisted of access to a single dipper (.1 mL) of 20% alcohol. The dipper remained in the raised position for 10 s, during which a light in the dipper aperture was illuminated. If a photobeam, directly in front of the dipper, was broken, the alcohol reward was considered “consumed”. On the first day of pretraining, alcohol was available on an FR 1 schedule (each response produced an alcohol reward). Each day, the dose of alcohol consumed was calculated and, if the subject consumed a dose above the criterion dose (>0.3 g/kg), then the ratio schedule was increased on the following day. After a subject consumed a dose above the criterion dose on an FR 4 schedule of reinforcement, subsequent sessions were conducted with a variable ratio (VR) schedule (modified from Fleschler & Hoffman’s [32] constant probability distribution), in which the number of required responses varied around an average value. Rats first responded on a VR 4, and the average ratio schedule increased by 2 on subsequent days if the dosing criterion was met. Throughout Pretraining, there were no programmed consequences for presses to the inactive lever, and the chain (alternative reinforcement manipulandum) was not available.

After subjects consumed a dose of alcohol above the criterion at a VR 10 schedule of reinforcement, they were assigned to a group (Sal or Nic; matched on pre-training alcohol consumption) and surgery was conducted (see below). Throughout the experiment, all sessions terminated after 60 min.

#### Surgery

Osmotic minipumps (model 2ML4; Alzet, Cupertino, CA), dispensing 60 μl of solution/day at a constant rate for 28 days (i.e., 3.0 mg/kg/day), were used for nicotine administration. Prior to implantation, pumps were filled with a liquid solution. For half of the subjects, nicotine solution (3.0 mg/kg free base per day) was used and for the other half of the subjects, saline alone was used. Rats were anesthetized with isoflurane and pumps were inserted into a subcutaneous pocket in the rat’s dorsal thoracic area via a small incision [33]. Rats were allowed two days to recover from surgery during which twice-daily injections of an NSAID analgesic (Flunixin Meglumine, 1.1 mg/kg, subcutaneous) and an antibiotic (Gentamicin, 2.0 mg/kg, intraperitoneal) were administered. Following recovery from surgery rats began alcohol self-administration (i.e., Baseline).

#### Baseline

During Baseline, a VR 10 schedule of reinforcement was in place on the target lever, and reinforcer deliveries consisted of access to a single dipper (0.1 mL) of 20% ethanol solution for 10 s. During reinforcer deliveries, the LED lights above the lever extinguished and the light in the dipper aperture was illuminated. There were no programmed consequences for presses to the inactive lever, and the chain (alternative reinforcement manipulandum) was not available.

#### Alternative reinforcement

Following Baseline, rats responded on the chain to earn access to chocolate pellets. A VR 4 schedule of reinforcement was in effect for 10 days. During the first two sessions of the phase, the first 10 reinforcers were available on an FR 1 to facilitate acquisition of chain pulling [34]. Reinforcer deliveries consisted of a single chocolate pellet. Following a pellet delivery the LED lights above the lever were extinguished and the light in the pellet/dipper aperture was illuminated for 10 s. There were no programmed consequences for presses to the target or inactive levers.

#### Resurgence testing

Following the Alternative phase, rats completed three sessions in which all reinforcement was suspended. There were no programmed consequences for responses to the levers or the chain.

### Data analysis

To first establish that the two-bottle choice procedure was successful in inducing consumption of alcohol, a linear mixed-effects model was conducted, using the lme4 package [35] in *R* [36]. For this analysis, the percentage of alcohol consumed (i.e., the amount of alcohol consumed divided by the total amount of liquid consumed) per day was the dependent variable and session was the sole independent variable. A random intercept of subject (rat) and random slope (session) were included because they were found to significantly improve the model. The significance of the predictor was evaluated using a Wald test via the car package [37], and the necessity of additional random effects was evaluated using likelihood ratio tests.

Next, the effects of nicotine on target responding were assessed across phases. To account for any individual differences in response rate, we calculated the proportion of baseline responding for each session during the subsequent phases. To calculate the proportion of baseline responding, the response rate (target responses / min) during each session of Alternative Reinforcement and Resurgence Testing was divided by the response rate during the last session of Baseline. If responding did not change from Baseline, the proportion of baseline responding would be equal to 1. If responding increased or decreased from Baseline, the proportion of baseline responding would be greater than or less than 1, respectively. Proportion of baseline responding was used as the dependent measure for the analyses that follow.

The effects of nicotine on target responding were analyzed across phases using linear mixed-effects modeling in *R* [36] using the lme4 package [35]. The initial model tested included Session, Phase, Group, and all of their interactions as predictors of target responding. This initial model included a random intercept of subject (rat) and no a-priori random slope effects. A three-way interaction between these variables was anticipated because the contingencies for target responding changed across phases, behavior subsequently shifted to conform to these new contingencies across sessions (more or less rapidly, depending on the phase), and thereafter, any effect of nicotine would most likely further moderate these differences. Significance of predictors and necessity of random effects were assessed as described above. Specific comparisons of target responding across phases and groups were conducted using the lsmeans package [38]. To clarify the nature of the three-way interaction, follow-up models were conducted within each experimental phase, including predictors of Group, Session, and their interaction. The random effects structure for these follow-up models was the same as that for the final model (see Results below).

Finally, we conducted two additional analyses to assess any additional relations in the data. First, we assessed the latency to the first target lever press during the first session of Resurgence Testing as a function of group membership with a Mann Whitney *U* test. Then, we assessed the correlation between alcohol consumption during the two-bottle choice procedure and the degree of resurgence observed by conducting a Spearman correlation on average g/kg consumed during the final week (i.e., 3 sessions) of two-bottle choice and degree of resurgence on the first day of resurgence testing (number of target responses on the first day of Resurgence Testing–number of target responses on the last day of Alternative Reinforcement).

## Results

**Fig 1:**
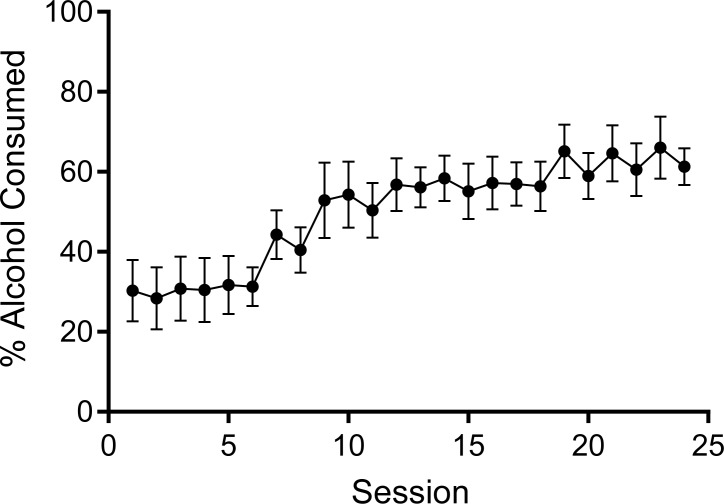
Percentage of alcohol consumed (i.e., ml of 20% alcohol / (ml of water + ml of 20% alcohol) averaged across all subjects for each session of the two-bottle choice procedure. Error bars represent standard error of the mean.

The two-bottle choice procedure produced escalation of alcohol intake across the 24 sessions using this procedure. Fig 1 shows session-by-session percent alcohol consumption during the two-bottle choice procedure. The increase in percent alcohol consumption was confirmed via a significant fixed effect of session on percent of alcohol consumption χ^2^ (1) = 24.43, *p* < .0001, such that percent of alcohol consumption increased as duration of exposure increased (*B* = 1.64, *SE* = 0.33). On the first day of exposure, rats overall showed a relatively low percentage of alcohol consumption (31%, *SE* = 6.5), which subsequently increased to 49% (*SE* = 4.70) and 69% (*SE* = 5.50) in sessions 12 and 24, respectively. Although the percent of alcohol consumption overall increased with session, there were individual differences in the extent to which alcohol consumption changed across sessions (random slope of session; χ^2^ (2) = 30.61, *p* < .0001).

**Fig 2:**
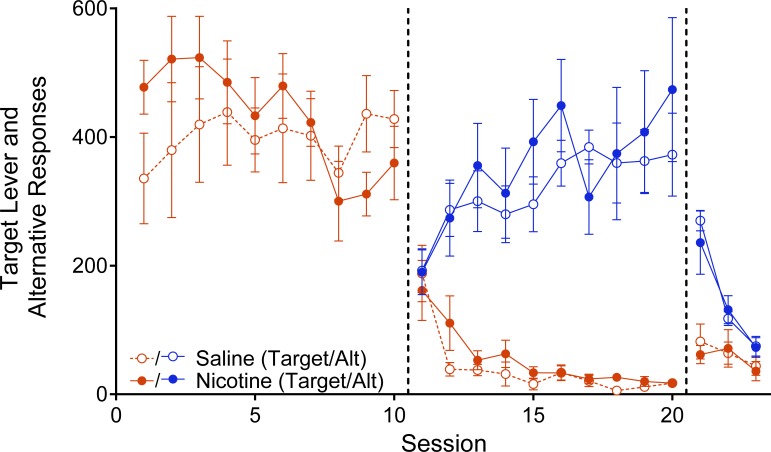
Average Target (red data paths) and Alternative (blue data paths) manipulanda responses plotted as a function of session across phases. Filled circles represent data for the Nicotine group and open circles represent data for the Saline group. Error bars represent standard error of the mean.

Fig 2 depicts the average number of responses on the target lever (red data path) and alternative reinforcement chain (blue data path) as a function of session for the Sal (open circles) and Nic (closed circles) groups, across each phase of the experiment. The average number of target responses per session was relatively high for both groups during Baseline (when responses on this lever produced alcohol), decreased during Alternative Reinforcement (when responses on this lever no longer produced alcohol and responses on a chain produced chocolate pellets), and increased during Resurgence Testing (when responses to both manipulanda were placed on extinction). Responding on the chain increased during Alternative Reinforcement (when responses on the chain produced chocolate pellets) and decreased during Resurgence Testing (when responses on the chain no longer produced chocolate pellets) at similar rates for both groups.

The number of inactive lever responses per session did not significantly increase for either group from the last session of Alternative Reinforcement (Nic: *M* = 12.17, *SEM* = 7.37; Sal: *M* = 11.17, *SEM* = 3.89) to the first session of Resurgence Testing (Nic: *M* = 20.67, *SEM* = 9.47; Sal: *M* = 10.00, *SEM* = 2.91). A 2 X 2 (Session X Group) mixed-model ANOVA performed on inactive lever responses between the last session of Phase 2 and the first session of Phase 3 revealed non-significant main effects of session *F*(1,10) = .618, *p* = .45, η_p_^2^ = .058 and group *F*(1,10) = .012, *p* = .526, η_p_^2^ = .041 and a non-significant Session X Group interaction *F*(1,10) = 1.073, *p* = .325, η_p_^2^ = .097. Thus, inactive lever responding did not increase when alternative reinforcement was removed, indicating that responding during Phase 3 was directed at the target lever, rather than the product of a general increase in responding induced by extinction of the alternative response.

The final linear mixed-effects model (referred to as “main model” henceforth for simplicity) included the addition of a random slope effect of session, which significantly improved the model, χ^2^ (2) = 10.59, *p* = .005. Responding shifted systematically as a function of session and phase. This result is evident in the main model (see Table 1) by significant main effects of Session (χ^2^ [1] = 15.03, *p* < .001) and Phase (χ^2^ [2] = 958.45, *p* < .001). The number of lever presses per session for the Sal and Nic groups, however, was not significantly different throughout the experiment. This result is illustrated by the lack of a main effect of Group (χ^2^ [1] = 1.86, *p* = .17). The interaction between Session, Group, and Phase was significant (χ^2^ [2] = 9.75, *p* = .008), however, which required follow-up analyses to understand. Thus, the main model was used to evaluate differences in responding across phases, but group differences and trends of responding within each phase are determined from follow-up models.

**Table 1:** Multilevel model results from the full final model.

Fixed Effects	β	S.E.
Intercept	328.03	25.47
Session	-22.96	4.96
Saline	92.05	36.02
Alternative Reinforcement	-333.27	31.76
Resurgence	-258.75	41.49
Session x Saline	27.58	7.01
Session x Alternative Reinforcement	9.74	5.95
Session x Resurgence	10.13	27.35
Saline x Alternative Reinforcement	-98.58	44.92
Saline x Resurgence	-78.57	58.68
Session x Saline x Alternative Reinforcement	-25.88	8.41
Session x Saline x Resurgence	-33.83	38.67
**Random Effects**	**Variance**	**S.D.**
Subject (Intercept)	864.40	29.40
Session	41.30	6.43
Residual	8761.70	93.60

During baseline, the two groups showed different trends in the number of responses per session across sessions (see Fig 1). The Nic group showed a downward trend in the number of target responses across Baseline sessions relative to the Sal group. This finding is evident in the follow-up model of responding in baseline (see Table 2) where a significant Session X Group interaction (χ^2^ [1] = 6.28, *p* = .01) was observed. Despite this difference in the trend of responding for alcohol, there were no differences in the number of responses per session between groups in any given session (all *p*s > .13).

**Table 2:** Follow-up model for baseline phase.

**Fixed Effects**	**β**	**S.E.**
Intercept	328.03	44.64
Session	-22.96	7.78
Saline	92.05	63.13
Session x Saline	27.58	11.00
**Random Effects**	**Variance**	**S.D.**
Subject (Intercept)	8495.10	92.17
Session	241.60	15.54
Residual	10014.90	100.07

From Baseline to Alternative Reinforcement, the main model showed a significant decrease in target responding across both groups (*t* [254] = 17.03, *p* < .001). Fig 1 shows that target responding decreased substantially from the end of Baseline to the beginning of Alternative Reinforcement for both groups. Within the Alternative Reinforcement phase, target responding decreased across sessions in both groups to a similar extent. This effect is evident in the Alternative Reinforcement follow-up model (see Table 3), which shows a significant main effect of Session (χ^2^ [1] = 53.73 *p* < .001), but no significant main effect of Group (χ^2^ [1] = 1.44, *p* = .223) nor a Session X Group interaction (χ^2^ [1] = 0.25, *p* = .61).

**Table 3:** Follow-up model for alternative reinforcement phase.

**Fixed Effects**	**β**	**S.E.**
Intercept	-5.24	13.01
Session	-13.22	3.74
Saline	-6.53	18.39
Session x Saline	1.70	5.28
**Random Effects**	**Variance**	**S.D.**
Subject (Intercept)	203.70	14.27
Session	55.30	7.44
Residual	2348.30	48.46

**Fig 3:**
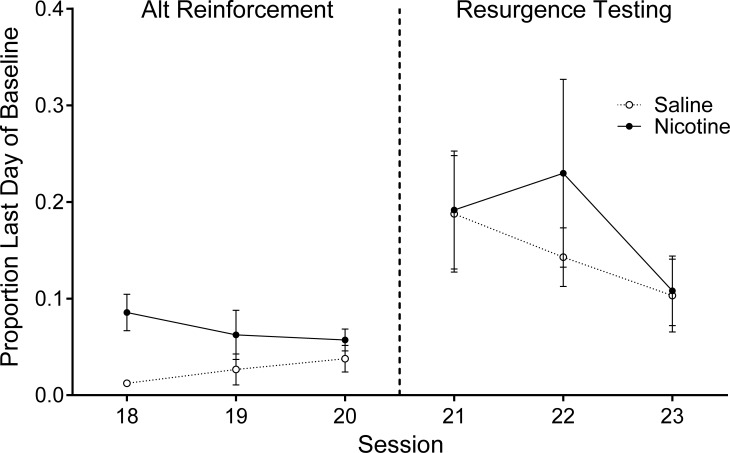
Average proportion of Baseline Target (alcohol) lever responses plotted as a function of session for the Nicotine and Saline groups. Data are plotted for the last three days of the Alternative Reinforcement Phase (left) and the three days of Resurgence Testing (right). Filled circles represent data for the Nicotine group and open circles represent data for the Saline group. Error bars represent standard error of the mean.

From Alternative Reinforcement to Resurgence Testing, target responding increased for both groups to a similar extent (see Fig 3). Results from the main model showed a significant increase in target responding across both groups (*t* [254] = -2.88, *p* = .004); however, there was no difference between groups in target responding on the first day of Resurgence testing (*t* [150] = -0.26, *p* = .80). These results are depicted in the first data point of the last phase in Fig 2. To further highlight this finding, Fig 3 depicts the proportion of baseline target responses as a function of session, for the last three days of Alternative Reinforcement and the three days of Resurgence Testing for the Sal (open circles) and Nic (closed circles) groups. There was an increase in proportion of baseline responding from the last day of Alternative Reinforcement to the first day of Resurgence Testing in both groups, but the increase was similar for both groups (i.e., there was no group difference in the degree of resurgence).

The two follow-up analyses also yielded no significant results. All rats responded on the chain at the beginning of the first session of Resurgence Testing. However, there was no group difference in the latency to the first response emitted (Nic: *Mdn* = 2.67 mins.; Sal: *Mdn* = 2.01 mins.) on the target lever during the first session of resurgence testing, *U* = 17.00, *p* = .937. There was also no correlation between the degree of resurgence (i.e., the number of target responses on the first day of Resurgence–the number of target responses on the last day of Alternative Reinforcement) and average consumption of alcohol for the last three days of the two-bottle choice procedure, *r*_*s*_(10) = .378, *p* = .227.

## Discussion

The results clearly illustrate that resurgence of alcohol seeking occurred in both the Nic and Sal groups. That is, in both groups, there was a significant increase in the number of target responses on the first day of Resurgence Testing relative to the number of target responses on the last day of Alternative Reinforcement. The findings for both groups in this experiment replicate the few prior studies that have shown resurgence of alcohol seeking [34,39].

This study is the first to investigate the effect of nicotine on resurgence of alcohol seeking. We did not find any evidence to support our hypothesis that nicotine augments relapse for alcohol seeking. There were no group differences observed across the entire study. Both groups responded on the target lever for alcohol to a similar degree during Baseline, both groups decreased responses on the target lever to a similar degree during Alternative Reinforcement, and both groups increased responses on the target lever during Resurgence Testing to a similar degree.

The results of the current study suggest that nicotine administration does not influence resurgence for alcohol seeking, but it is also possible that that the methodology employed hindered our ability to detect an effect of nicotine on resurgence of alcohol seeking. Osmotic minipumps have been used extensively to investigate the effects of chronic drug exposure. They are a useful tool that overcomes many challenges that are encountered with other drug delivery techniques (e.g., stress, conditioned drug effects from regular injections, costly equipment, lengthy training of staff, etc.). Osmotic minipumps have been used successfully to investigate the relation between continuous nicotine exposure and alcohol self-administration [40].

However, Brynildsen et al. [41] argue that continuous nicotine delivery, via osmotic minipumps, may not adequately model human nicotine intake. Human smokers (and “vapers”) have an intermittent pattern of nicotine intake throughout the day and prolonged withdrawal throughout the night. Brynildsen et al. argue that this pattern of intake allows nicotinic acetylcholine receptors to return to a fully active state between smoking episodes [42]. The intermittency of nicotine exposure in human smokers is thought to be critical to the addictive nature of the drug and may also play an important role in the reward-enhancing effects attributed to the drug. By using a continuous nicotine delivery method in the current study, the reward-enhancement of alcohol by nicotine may have been affected, as the reward-enhancing properties of nicotine are mediated by these receptors as well [43].

The decreasing trend in target responding for the Nic group during Baseline (as opposed to stable responding for Sal group during this phase; see Fig 1) may be indicative of desensitization of nicotinic acetylcholine receptors. The initial elevation in target lever responding for the Nic group relative to the Sal group suggests that nicotine may have made alcohol more reinforcing, but this effect waned across sessions to the point that the Nic group actually responded for alcohol slightly less than the saline group by the end of this phase. This decreasing trend in target responses may be the product of nicotinic acetylcholine receptor desensitization and directly related to our decision to use continuous delivery of nicotine as opposed to intermittent delivery of nicotine. Future research should investigate the effect of intermittent nicotine exposure on resurgence of alcohol seeking to assess whether or not the same results are observed.

In summary, we investigated the effect of continuous nicotine exposure on resurgence of alcohol seeking. We predicted and found resurgence of alcohol seeking in both the Nic and Sal groups. We further predicted, however, that nicotine would augment resurgence of alcohol seeking relative to a saline control group. We found no evidence to support this latter hypothesis. However, this null result may be due to the continuous drug delivery method that was chosen, and different results may be observed if nicotine administration was conducted intermittently as opposed to continuously.

## Supporting information

S1 Dataset In the supporting information file, there are 7 columns. The first column is group assignment. The second column is subject ID. The third column is phase. The fourth column is session number. The fifth column is total grams of alcohol drunk during the session. The sixth column is grams of alcohol drunk per kilogram in the session. The seventh column is the number of active lever responses per session.
(XLSX)
